# Peirce's revenge on the Chinese Room

**DOI:** 10.3389/fpsyg.2026.1900073

**Published:** 2026-07-30

**Authors:** Evgeny V. Loginov, Vadim V. Vasilyev, Anton V. Kuznetsov, Dmitry B. Volkov, Artem P. Besedin, Andrew V. Mertsalov

**Affiliations:** 1 Faculty of Philosophy, Lomonosov Moscow State University, Moscow, Russia; 2 The International Center for Consciousness Studies, Messina, Italy

**Keywords:** Chinese Room, large language models, linguistic understanding, Peirce, Searle

## Abstract

Since its publication in 1980, John Searle's Chinese Room argument has remained one of the central objections to computational theories of mind and machine understanding. In this paper, we argue that the force of the Chinese Room depends on an implausibly impoverished conception of linguistic interaction: namely, the assumption that a system capable of sustaining unrestricted Turing-level communication could nevertheless remain completely isolated from the pragmatic dimensions of language. Using a Peircean distinction between icons, indices, and symbols, we show that ordinary linguistic interaction involves iconic, indexical, temporal, rule-governed, and pragmatically embedded communicative practices that Searle cannot fully allow inside the Chinese Room without either permitting at least partial semantic understanding or artificially restricting the scope of the test. We then generalize this dilemma beyond classical symbolic AI by introducing the Chinese Token-Training Room, a philosophical analog of recent “stochastic parrot” objections to large language models. We argue that such objections must either restrict the system in ways that make unrestricted linguistic competence doubtful, or allow forms of pragmatic embedding that weaken the intuition of total semantic opacity. We conclude that neither the original Chinese Room nor its token-training analog establishes that functional or computational organization is in principle insufficient for conscious semantic understanding; rather, they show at most that radically impoverished forms of isolated symbol or token manipulation are insufficient.

## 1 Introductory remarks

In the contemporary era of rapidly developing artificial intelligence, it is useful to revisit the classical dispute between biological naturalism and functionalism about consciousness, since this debate may shed light both on the nature of consciousness in general and on the possibility of conscious AI in particular. In contemporary philosophy of mind, functionalism is usually understood as the view according to which what is essential for consciousness is the functional or computational properties of a system rather than the particular physical substrate realizing them (Sellars, 1952; Putnam, 1960; Dennett, 1969; Searle, 1980, 1984, 1990). By contrast, various forms of biological naturalism and other ‘meat-based' approaches — recently reconsidered, among others, by Ned Block (2025) — maintain that consciousness may depend not only on computational organization but also on specific biological or subcomputational mechanisms implementing the relevant functions. Put briefly, the difference between these positions is that computational functionalism assumes that consciousness is substrate-independent, whereas biological naturalism denies such substrate independence.

We should note, however, that the opposition between computational functionalism and biological naturalism does not exhaust the contemporary landscape of positions. Some philosophers defend intermediate views. Anil Seth, for example, proposes a form of ‘substrate flexibility', according to which consciousness may be realizable in different physical media, though not necessarily in arbitrary ones (Seth, 2025). On such a view, consciousness is neither strictly tied to biological tissue nor completely substrate-independent in the strong functionalist sense. There are also more unusual positions, such as Reza Negarestani's quasi-Hegelian functionalism, which interprets mindedness less in terms of particular physical realization and more in terms of participation in inferential and normative structures (Negarestani, 2018). A recent powerful argument also suggests that both camps overstate what the current evidence can actually support (McClelland, 2025). However, in the present paper we will largely bracket such intermediate positions and focus primarily on the opposition between biological and functionalist approaches to consciousness — particularly as this opposition bears on the relationship between advanced linguistic competence and the possibility of genuine understanding or consciousness.

Before proceeding, a clarification is needed concerning the relation between consciousness and understanding. In contemporary philosophy of mind, following distinctions such as Block's distinction between access consciousness and phenomenal consciousness, or Chalmers's distinction between psychological and phenomenal consciousness, many philosophers may dissociate these notions. They may allow for a form of unconscious or non-phenomenal understanding, if by this they mean a functional or access-level capacity that lacks phenomenal or qualitative character. Searle's framework is different. For him, consciousness is essentially subjective and qualitative, and unconscious mental states are intelligible only in relation to their capacity to become conscious. In the context of the Chinese Room, the relevant notion of understanding is therefore not a merely functional or behavioral capacity. Searle's argument turns on the contrast between the manipulation of uninterpreted symbols and the conscious grasp of meaning. Thus, while consciousness is not reducible to understanding, the kind of understanding at issue in Searle's argument is conscious semantic understanding.

## 2 Historical background

If we look further back into the history of philosophy, functionalist intuitions and objections to them may be said to arise almost simultaneously with philosophy itself. An early, still largely metaphorical formulation appears already in Plato's *Phaedo* (86a−87a), where the soul is compared to harmony and the body to a lyre. The underlying intuition is functionalist in spirit because the harmony is not identified with any particular material component of the lyre, but rather with the organized relational structure produced by the arrangement of its parts. Plato, however, rejects this analogy because it conflicts with his doctrine of recollection and because the soul appears to govern the body, whereas harmony does not govern the lyre but rather depends upon it. A structurally similar line of thought reappears in Leibniz's famous mill argument in the *Monadology* (§17), where he invites us to imagine a machine enlarged to the size of a mill and entered from within; according to Leibniz, one would find only mechanical interactions among parts and never anything resembling perception or thought. Leibniz's argument may thus be interpreted as an early attempt to deny that purely functional or mechanical organization is sufficient for consciousness. It is also likely that the argument was directed, at least in part, against Hobbes's influential claim that reasoning is fundamentally a form of computation or reckoning, and that thought therefore consists in nothing more than mechanically manipulable operations.

One of the earliest modern thought experiments directed against functionalist intuitions was Anatoly Dneprov's *The Game* (Dneprov, 1961). In Dneprov's story, thousands of mathematicians collectively simulate the operations of a computer translating Portuguese into Russian while none of the participants understands either the overall task or the semantic content of the translated sentence. The thought experiment is intended to show that successful formal information processing may occur without genuine understanding, even when the system as a whole successfully performs an apparently intelligent linguistic task. A related line of criticism was developed by Hubert Dreyfus, who from the mid-1960s onward argued that machines would never genuinely think because human intelligence depends on embodied, context-sensitive, and skillful engagement with the world rather than on the formal manipulation of discrete symbols (Dreyfus, 1965, 1992; Dreyfus and Dreyfus, 1986). However, Dreyfus's critique differs from Dneprov's in that many of his claims concerned specific machine capacities and were later empirically weakened by developments in AI, while his deeper argument relied on a Heideggerian-phenomenological conception of human being-in-the-world that need not be accepted by functionalists. Ned Block's ‘Chinese Nation' thought experiment develops a similar anti-functionalist intuition on a much larger scale. Block asks us to imagine an entire nation of people collectively simulating the functional organization of a human brain by communicating with one another in ways corresponding to neuronal interactions. Even if such a system successfully reproduced the functional organization of a conscious brain, it still seems deeply implausible that a unified phenomenal consciousness would thereby emerge at the level of the nation as a whole (Block, 1978).

This style of anti-functionalist intuition can be pushed even further by modifying the Chinese Nation scenario. Suppose we possess a complete and perfectly accurate record of the contents of David Hume's mind at the precise moment when he was drinking claret and reflecting on the problem of induction. Now imagine an enormous and densely populated planet whose inhabitants collectively simulate, neuron by neuron, the activity of Hume's brain during that moment. However, these people are extremely lazy: each individual performs no more than a single action per day. In principle, this need not prevent the realization of the relevant functional organization. The process that took only a few seconds in Hume's brain would simply require several years on the Lazy Hume Planet. Nevertheless, it seems highly implausible that somewhere on this planet there would thereby arise the taste of wine together with Hume's conscious reflection on induction. Functionalists might reply that phenomenal consciousness in such a case would consist of discrete ‘flashes' occurring during moments of information exchange. Yet this response itself appears problematic, since such isolated ‘flashes' would no longer constitute the temporally unified conscious experience originally instantiated by Hume.

However, even in the case of the Lazy Hume Planet, Block's argument ultimately remains more of an intuition pump than a decisive refutation of functionalism. Some may find it intuitively obvious that such a planet could not possess consciousness, whereas others may deny this. A functionalist may simply reply that, even if the Lazy Hume Planet appears deeply counterintuitive, this merely reflects one of the surprising consequences of the theory that must nevertheless be accepted if the theory itself is correct (Chalmers, 1996, p. 249). After all, when we examine the structure of a modern computer, we may wonder how a mere collection of mechanisms and electrical circuits could possibly perform highly sophisticated mathematical operations, since mathematics itself appears to require genuine creative reasoning. Yet few today would conclude from this intuition that machines are incapable of mathematical thought simply because such reasoning initially appears strange or counterintuitive. The same may ultimately be true of consciousness and the brain itself.

John Searle's Chinese Room argument develops and radicalizes earlier anti-functionalist intuitions. Like Leibniz's mill argument, it attempts to show that purely formal processes are insufficient for genuine understanding; like Dneprov's *Game*, it argues that successful linguistic performance may occur without semantic comprehension; and like Block's Chinese Nation, it challenges the idea that functional organization alone is sufficient for consciousness. The argument is also closely connected to Alan Turing's famous proposal that intelligence may be operationally tested through successful linguistic interaction. In the Turing Test, a machine counts as intelligent if, during unrestricted textual conversation, a human interlocutor cannot reliably distinguish it from another human being (Turing, 1950). Searle's Chinese Room is intended precisely as a criticism of this criterion: according to Searle, a system may successfully pass the Turing Test while nevertheless lacking any genuine understanding of the language it uses. However, Searle's thought experiment introduces an important methodological innovation. Instead of merely asking us to imagine an externally observed system, Searle invites the reader to place himself inside the system and personally execute the computational procedure. Whereas Block's argument primarily appeals to common-sense intuitions about the implausibility of a collective consciousness emerging at the level of an entire nation, Searle appeals to the first-person intuition that one could follow purely formal rules for manipulating symbols without thereby understanding anything. The central force of the argument, therefore, lies in the apparent gap between syntactic symbol manipulation and semantic understanding as experienced from the perspective of the agent implementing the program.

Abstracting from the details of the thought experiment, Searle's argument may be presented in a very simple form:

(1) Computer programs are purely syntactic;
(2) Syntax is not sufficient for semantics;
(3) Understanding is semantic;
(4) Therefore, computer programs are not sufficient for understanding.

This line of reasoning is closely related to what Harnad later called the symbol grounding problem: the problem of explaining how the semantic interpretation of a formal symbol system can be intrinsic to the system rather than parasitic on meanings already present in the minds of external interpreters (Harnad, 1990, 1993; Bringsjord and Noel, 2002; Taddeo and Floridi, 2005).

Some commentators have even jokingly suggested that cognitive science could be redefined as “the ongoing research program of showing Searle's Chinese Room Argument to be false” (Harnad, 2002, p. 295). As Searle himself modestly remarked in a 2015 interview, “I have no idea of the influence of the Chinese Room Argument. It is obviously right, and I assume in the end, people will recognize that” (Searle, 2015, p. 53).

Over the decades since the publication of the Chinese Room argument, virtually every aspect of Searle's original paper has been challenged and subjected to criticism. Among the most influential objections is the Systems reply, according to which, even if the person inside the room does not understand Chinese, the larger system consisting of the person, the rulebook, and the room as a whole may nevertheless possess understanding. Searle's response to this objection has several layers.

First, he insists that the mere addition of the rulebook and the pieces of paper cannot by itself generate understanding: if the person in the room does not understand Chinese, and the papers containing the rules plainly do not understand Chinese either, then it is unclear how the combination of two non-understanding components could suddenly amount to understanding.

Second, Searle strengthens this point by asking us to suppose that the person memorizes all the rules and internalizes the entire system. In that case, the room disappears entirely: the person no longer consults external instructions but continues to manipulate Chinese symbols purely mechanically from memory. According to Searle, even under these conditions no understanding appears. The resulting scenario resembles xenoglossy — the purported paranormal phenomenon in which a person suddenly begins to produce meaningful linguistic behavior in a language he has never learned. The individual from the Chinese Room receives Chinese symbolic inputs and immediately produces appropriate Chinese symbolic outputs, while still allegedly understanding none of the symbols involved.

While this reply is powerful, it also may create pressure on Searle's own position. The Systems reply may seem counterintuitive, but many genuine cognitive systems are counterintuitive when described at the wrong level. Imagine a native Chinese speaker whose neurotransmitters cease to transmit signals between neurons and are replaced by a tiny ‘Searle demon' that runs between the neurons and transmits electrical impulses exactly as the neurotransmitters would have done. If the demon perfectly preserves the causal organization of the brain, the person's behavior and linguistic capacities remain unchanged. Searle seems committed to saying that the person still understands Chinese. But then the relevant subject of understanding is not the demon, any more than it is an isolated neurotransmitter or neuron; it is the organized system as a whole. This suggests that asking whether the isolated man inside the Chinese Room understands Chinese may itself involve a category mistake: understanding may belong not to a proper part of the system, but to the system at the appropriate level of organization.

A possible reply on Searle's behalf would emphasize an important asymmetry between the ‘Searle demon' case and the xenoglossy-style Chinese Room scenario. The native Chinese speaker whose neurotransmitter functions are replaced by the demon should indeed still count as understanding Chinese — and both the functionalist and the biological naturalist are likely to agree about this. However, unlike the isolated symbol manipulator in the Chinese Room, the Chinese speaker with the demon in his brain remains continuously embedded in ordinary causal interaction with the world. His linguistic behavior is integrated with perception, action, memory, bodily activity, and ongoing pragmatic engagement with the environment. By contrast, the xenoglossy-like figure produced by the Chinese Room remains connected to the world only through the formal manipulation of symbols. Precisely because of this radical pragmatic isolation, we are inclined to deny that genuine understanding is present in the Chinese Room case even if the outward linguistic behavior appears correct. Although the Systems reply can be further developed in a number of sophisticated directions, we will not pursue those strategies in the present paper (Dennett, 1991, p. 435–440; Cole, 2026).

Other influential criticisms include Paul and Patricia Churchland's claim that Searle illegitimately infers limits on future AI from our current ignorance concerning the neurobiological basis of consciousness (Churchland and Churchland, 1990), as well as David Chalmers's implementation-based objection, according to which Searle conflates a formal specification of a process with its concrete physical realization (Chalmers, 1996, p. 327–8). The Churchlands' response does not so much analyze the internal logic of Searle's argument as oppose scientific optimism to what they interpret as excessive pessimism concerning artificial intelligence. Yet Searle himself is not opposed to scientific progress in neuroscience or cognitive science; rather, he denies that future scientific discoveries will establish an identity between consciousness and purely functional substrate-independent states, because he takes no such identity to exist. Similarly, Chalmers's objection arguably misses the target of the Chinese Room argument, since Searle's scenario concerns not a merely abstract formal structure but precisely its implementation — that is, a situation in which an agent actually follows the relevant instructions and manipulates symbols in accordance with the program.

As Perconti and Plebe have recently observed, the contemporary revival of interest in the Chinese Room argument is closely connected to the recent boom in artificial intelligence and especially to the emergence of increasingly sophisticated large language models. Yet this renewed attention has not substantially enriched the debate from the standpoint of argumentation: discussions still largely revolve around the original intuition that symbol manipulation and genuine understanding are fundamentally different kinds of processes (Perconti and Plebe, 2023). In this paper, we develop a line of criticism of the Chinese Room argument that has received comparatively little attention in the existing literature. Our strategy does not consist in attempting to show, contrary to Searle, that functional properties are sufficient for consciousness. Rather, we aim to show that Searle fails to provide sufficient grounds for concluding that functional properties are insufficient for consciousness. However, our reason for rejecting the force of the Chinese Room argument differs from the orthodox functionalist response according to which genuine understanding or consciousness would necessarily emerge within the system. Our strategy is instead to argue that the original Chinese Room, as described by Searle, would likely be incapable of successfully passing an unrestricted Turing Test and therefore cannot straightforwardly function as a thought experiment demonstrating that a system capable of passing the Turing Test would nevertheless obviously lack understanding. In short, we aim to show that it is highly implausible that the original Chinese Room could genuinely sustain unrestricted Turing-style interaction. If this is correct, the force of Searle's argument is significantly weakened.

## 3 The Peircean revenge

In the course of our work, we developed several different directions in which such a critique of the Chinese Room argument might proceed. Since a mere proliferation of disconnected objections would risk obscuring the underlying structure of our position, we have chosen — to a considerable extent arbitrarily — to organize our discussion through Peirce's theory of signs. The central idea of our argument does not depend either on Peirce's broader philosophical framework or on the more technical details of his semiotics. Nevertheless, our objections collectively suggest that, alongside the distinction between syntax and semantics that lies at the heart of Searle's Chinese Room argument, there also exists a pragmatic dimension of language whose neglect may ultimately undermine Searle's project itself — an irony made especially striking by the fact that Searle is one of the major theorists of speech *acts*. Moreover, since the familiar distinction between syntax, semantics, and pragmatics ultimately derives from the Peircean tradition through figures such as Charles Morris, one might say that what follows constitutes, at least in part, a kind of ‘Peircean revenge'. While Peirce's semiotics has occasionally been invoked in support of Searle's position (Brown, 2002), we argue that a closer consideration of iconic, indexical, and symbolic dimensions of linguistic practice actually undermines the Chinese Room thought experiment when it is required to model unrestricted Turing-level communication.

Peirce's theory of signs can be presented in many different ways and developed with varying degrees of technical sophistication (Peirce, 1984, p. 49–58; Short, 2007). For our purposes, however, a very simplified exposition will suffice. Consider the statement: ‘This thing is black'. From a Peircean perspective, the first element we may isolate here is a certain *quality* — blackness. Yet a quality becomes intelligible only against the background of other possible qualities that function as its correlates; in other words, we must grasp the system of *relations* within which the relevant blackness is situated. At the same time, we must also be capable of synthesizing the reference to the quality with the reference to its correlate, thereby identifying some *interpreting principle* connecting the two. This simple line of thought underlies Peirce's triad of Firstness, Secondness, and Thirdness — roughly corresponding to quality, relation, and interpretant.

When these categories are applied to signs, we may study signs with respect to their intrinsic qualities (Firstness), their relations to objects (Secondness), and their interpretive or practical effects (Thirdness). Focusing specifically on the relation between signs and things, Peirce distinguishes icons, indices, and symbols. Icons are signs connected to their objects through qualitative resemblance; indices are signs connected to their objects through some real or causal relation; and symbols are signs connected to their objects through conventional rules or socially established practices. Our central idea is that ordinary computational systems capable of passing the Turing Test can freely employ iconic, indexical, and symbolic resources, whereas Searle, while manually implementing the program inside the Chinese Room, cannot plausibly allow himself equivalent forms of sign use without thereby weakening the original intuition of the argument.

### 3.1 Icons

Iconic signs, roughly speaking, are signs whose relation to their objects is grounded in resemblance. A portrait, for example, is an icon of the person it depicts. Harnad's grounding proposal already assigns a foundational role to iconic representations, understood as nonsymbolic analogs of sensory projections. Our use of iconicity differs from Harnad's, since we focus not primarily on internal sensory representations but on iconic and quasi-iconic elements within communicative practice itself (Harnad, 1990).

Let us now return to Searle inside the Chinese Room. According to the conditions of the thought experiment, Searle does not know Chinese; moreover, it is essential to the argument that he does not come to understand Chinese in the course of executing the program. Otherwise, the thought experiment would show precisely the opposite of what Searle, as the philosopher constructing the scenario, intends to show. Yet there are many signs that Searle could plausibly begin to understand under these very conditions. Suppose, for example, that the examiner sends him, in Chinese, a sequence of numerals — 1, 2, 3, … — and asks him to continue the series. Given the visual form of the Chinese numerals for one, two, and three, it is highly plausible that Searle, as an English speaker, would infer on his own that the next answer should be four. Moreover, once he consults the rulebook and discovers the corresponding symbol for four — which no longer visually resembles the number four for an English speaker — he will thereby have acquired at least a partial semantic understanding of one Chinese sign.

Before proceeding, we should clarify what we mean by “partial semantic understanding.” We do not mean merely a new behavioral disposition, statistical sensitivity, or externally describable competence. This phenomenon is familiar from ordinary linguistic experience. A person may read a text in a language he does not know and still recognize isolated words, names, numerals, diagrams, emoticons, typographical forms, or borrowed expressions. An English speaker reading Greek, for instance, may fail to understand Greek while still recognizing λóγoς as connected with “logos,” or may identify mathematical symbols, proper names, arrows, dates, or visual patterns in the text. Such recognition is not full linguistic comprehension, but it is also not semantic emptiness. From the subject's own point of view, some signs cease to be wholly opaque marks and begin to appear as signs of something. This is the sense in which we will speak of partial semantic understanding below. It is local, fragmentary, and compatible with massive ignorance of the language as a whole. At the same time, we are not appealing to the trivial fact that the person in the Chinese Room, being a human subject, inevitably has thoughts, associations, or background understanding of something. Such generic mental activity is not a problem for Searle, since it does not amount to understanding the Chinese signs sent by the examiner. Our claim is stronger: in the kinds of cases we will discuss—such as numerals and the further examples developed below, the subject may come to understand something that is directly relevant to the very Chinese signs he is processing. But it is still semantic in the sense relevant to Searle's argument, because it interrupts the first-person intuition of total meaninglessness on which the Chinese Room depends. A defender of Searle may, of course, redescribe such cases externally as associative learning or pattern sensitivity. But that reply changes the dialectical situation. Searle's argument does not rest merely on the possibility of an external syntactic description of the person's behavior; it rests on the claim that the person implementing the program can know, from the inside, that no understanding is present. Once iconic, indexical, or otherwise pragmatically transparent elements enter the exchange, that claim becomes unstable: the person may still fail to understand Chinese, but he no longer fails to understand anything at all.

We should also clarify the methodological status of the examples that follow. They are not intended to function as intuition pumps in the same sense as Block's Chinese Nation or similar thought experiments. Our appeal is not to a special philosophical intuition about whether some counterintuitive system really understands or is conscious. Rather, the relevant point is a more ordinary fact about natural language and communicative practice: signs can carry semantic information through iconic resemblance, indexical anchoring, conventional use, visual organization, and metalinguistic structure. The cases discussed below are meant to show that, once such resources are admitted into the Chinese Room, the claim of total semantic opacity becomes difficult to sustain; if they are excluded, the room no longer models unrestricted linguistic interaction.

With these clarifications in place, we can return to the numeral case and note an obvious limitation of the example. Searle could, of course, replace Chinese with a language or writing system in which no such accidental iconic transparency arises. For instance, one might imagine the room operating with Mongolian square script, or with some other unfamiliar notation in which the visual form of the signs provides no usable clue to their meaning for English speakers. In itself, such a move is perfectly legitimate. However, the Chinese Room is supposed to model a system capable of unrestricted linguistic competence, and therefore it should, in principle, be able to operate with arbitrary natural languages — including languages and communicative practices containing varying degrees of iconic structure. Thus, the example of numerals does not by itself refute the Chinese Room argument. It merely illustrates a more general point: insofar as the input may contain iconic elements, Searle cannot always prevent some degree of semantic access; but insofar as such elements are excluded by stipulation, the range of permissible Turing-style interaction is correspondingly narrowed.

A critic may object that language is not identical with a writing system, and this is entirely correct. If the inputs to the Chinese Room consisted purely of arbitrary sounds, then the iconic objection would lose much of its force. However, natural languages are rarely completely free from iconic or quasi-iconic elements even at the phonetic level. Many languages contain onomatopoeia, phonetic borrowings, and internationally recognizable expressions. For example, if the discussion in Chinese concerned Coca-Cola, an English speaker would very likely grasp at least the general topic of the exchange despite not knowing Chinese. Likewise, words such as ‘coffee', ‘pizza', or even ‘logic' and ‘humor' are often borrowed across languages and retain enough phonetic similarity that, when pronounced by a Chinese speaker, they can still be understandable to English speakers by ear. Searle could respond by stipulating that references to brands, proper names, borrowed words, and other internationally recognizable expressions are forbidden. Yet this move once again narrows the range of permissible linguistic interaction.

Moreover, similar difficulties can reappear whenever languages are represented through more familiar writing systems. If Chinese were transliterated into the Latin or Cyrillic alphabet (such transliterations already exist and work quite successfully), there would be a substantial chance that Searle would recognize at least some internationally widespread words and expressions. The broader point, therefore, is not that iconicity guarantees understanding, but that ordinary linguistic practice contains many partially transparent elements that continuously threaten the radical semantic opacity presupposed by the original Chinese Room scenario.

Another difficulty arises from the use of graphic signs such as emojis. Suppose the examiner writes something like “I 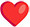 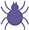,” meaning that she loves spiders. Searle could, of course, stipulate that such signs are not allowed in the exchange, but this would again restrict the range of communicative resources available in the test and thereby make it less plausible that he can do everything an ordinary machine capable of passing the Turing Test can do.

More importantly, the difficulty cannot be eliminated simply by replacing Chinese with another language or script. In any written language, and under virtually any writing system, it remains possible to introduce iconic elements through visual arrangement itself: shaped poems, calligrams, diagrams, arrows, spatial layouts, or other graphic devices. An examiner could, for example, present Searle with an experimental visual poem in the tradition of Lewis Carroll, Guillaume Apollinaire, Chen Li (陳黎), or Dmitri Prigov, where aspects of meaning are conveyed not only through lexical content but also through the spatial organization of the text itself. These cases suggest that iconicity is not merely an accidental feature of some familiar signs, but a recurrent dimension of written communication. Thus, Searle faces a dilemma: either he allows such iconic or quasi-iconic resources, in which case the person in the room may acquire at least partial semantic access to the exchange, or he excludes them by stipulation, in which case the Chinese Room no longer models unrestricted Turing-style linguistic competence. Dennett offers a similar iconic example (2014, p. 327).

The examples discussed above differ in force and philosophical significance, but they all point in the same direction. Existing computational systems, including systems available long before contemporary large language models, can easily participate in such forms of communication. Our claim is not that this by itself demonstrates the presence of understanding or consciousness, much less phenomenal consciousness, in such systems. Rather, the problem is that Searle cannot straightforwardly allow these communicative resources into the Chinese Room scenario while preserving the original anti-functionalist intuition. Either iconic or quasi-iconic elements are permitted, in which case the person inside the room may gradually begin to grasp significant aspects of the exchange, and the thought experiment starts to suggest the opposite of what Searle intended to show; or such elements are excluded by stipulation, in which case the conversational and linguistic capacities of the Chinese Room become substantially more restricted than those of ordinary computational systems. But once this restriction is introduced, the central force of the original argument is correspondingly weakened, since the Chinese Room no longer plausibly models unrestricted Turing-level linguistic competence.

### 3.2 Indices

Indexical signs are signs whose relation to their objects is not based primarily on resemblance, as in the case of icons, but on a real contextual connection. In ordinary language, indexical expressions include terms such as “I,” “this,” “that,” “here,” and “now.” What these expressions have in common is that their semantic value cannot be fixed independently of the context of utterance, that is, independently of who is speaking, where the utterance occurs, and when it occurs. Indexicals are commonly divided into several types. Temporal indexicals include expressions such as “now,” “yesterday,” “tomorrow,” and “today,” which locate events relative to the time of utterance. Spatial or locative indexicals include expressions such as “here,” “there,” “this place,” “right in front of you,” “to the left,” or “nearby,” which locate objects relative to the speaker's position. The word “I” refers to whoever is speaking; “here” refers to the place of the utterance; “now” refers to its time; “this” and “that” require some contextual relation of demonstration, attention, or spatial-temporal orientation. In this sense, indexicals acquire their semantic content through contextual anchoring rather than through context-independent lexical meaning alone.

We distinguish two kinds of indexical expressions that may create difficulties for Searle's argument. The first concerns spatial or perceptual indexicals, whose interpretation depends on the system's relation to its immediate environment; the second concerns temporal indexicals, whose interpretation depends on the system's ability to locate itself within the unfolding time of the exchange.

The first type of indexical difficulty concerns perceptual or spatial indexicals. Consider a simple example. Before the experiment begins, the examiner enters the Chinese Room and places a can of Coca-Cola on the table in front of Searle. The dialogue then starts, and the examiner asks in Chinese: “What is directly in front of you?” The problem is immediate. If the original Chinese Room is supposed to operate purely syntactically, then the correct answer — “a can of Coca-Cola” — must already somehow be encoded within the program. But how could the author of the program have known in advance which particular object the examiner would place in the room? The program would effectively need to anticipate an open-ended and indefinitely variable environment.

One possible response would be to insist that the relevant answer was already written into the rulebook. Yet this makes the thought experiment deeply implausible. The system would require something close to an omniscient program capable of anticipating arbitrary future contextual states. Worse still, once the examiner becomes aware of the structure of the program, nothing prevents him from changing the object and thereby immediately exposing the limitations of the system. A critic might reply that the room could simply answer that it does not know, thereby imitating an ordinary feature of natural linguistic interaction. However, a systematic inability to answer questions of this kind would itself reveal an important limitation in the system's linguistic competence. A conversational agent that regularly fails whenever communication depends on immediate perceptual or contextual grounding would hardly count as possessing unrestricted Turing-level language ability. In this sense, unrestricted Turing-style interaction cannot plausibly be sustained through purely syntactic resources alone.

Searle's natural response is to modify the original scenario by introducing what he himself calls the Chinese Robot. Instead of remaining in complete perceptual isolation, the room is connected to cameras, sensors, and other input devices. When asked “What is in front of you?”, Searle consults the rulebook, activates the relevant sensor system, receives some formally encoded information, and outputs the corresponding Chinese answer. At first glance, this appears to preserve the force of the original argument: Searle still manipulates symbols without understanding Chinese.

However, this modification fundamentally changes the structure of the thought experiment. In the original Chinese Room, Searle was supposed to know and execute the entire program. But once the room is connected to perceptual systems, the program no longer consists merely of formal relations among Chinese symbols. It now also includes systematic correlations between symbols and perceptual states generated by cameras, sensors, and interactions with the external world. If Searle internalizes the entire program, he will gradually begin to understand at least some of these correlations. He may not thereby become an ordinary fluent speaker of Chinese, but he can begin to grasp that certain groups of symbols correspond to visual objects, spatial orientations, movements, or perceptual events. In other words, the addition of sensorimotor grounding introduces precisely the sort of semantic relation between symbols and world that the original Chinese Room was designed to exclude.

The crucial point, therefore, is that the Chinese Room and the Chinese Robot are not equivalent systems. The original room preserves the intuition of purely syntactic symbol manipulation only because it is radically isolated from the world. But precisely for this reason, it is doubtful that it could pass an unrestricted Turing Test. The robotized version may overcome this limitation, yet it does so by introducing perceptual and pragmatic relations that partially undermine the original anti-functionalist intuition. Moreover, once Searle moves from the Chinese Room to the Chinese Robot, he faces a dilemma analogous to the one discussed above. If he has access to the entire program, then he can in principle discover systematic correlations between the robot's camera inputs, sensor data, and Chinese symbols, thereby acquiring at least a partial understanding of the language. If, on the other hand, he is denied access to those parts of the program and manipulates only a restricted subset of symbols, then he is no longer implementing the entire system. In that case, the Chinese Robot ceases to be equivalent to the Chinese Room, since crucial components of the cognitive architecture have been placed outside the scope of the thought experiment. Either Searle gains some degree of semantic access to the system, or the analogy between the Chinese Room and the Chinese Robot breaks down. In both cases, the force of the original argument is substantially weakened.

The second type of indexical difficulty concerns temporal indexicals and what might be called the problem of internal time. Unlike the Coca-Cola example, this objection does not depend on any external object being introduced into the room. Even if the Chinese Room is placed in complete sensory isolation, there still remain certain temporally indexed facts that arise during the conversation itself and cannot plausibly be specified in advance by the program. Consider questions such as: “How long has it been since the beginning of the conversation?”, or “Which interval was longer: the pause before my previous question or the pause before this one?” These questions do not require perceptual access to the external world; rather, they require the system to situate itself within the temporal unfolding of the dialogue itself. A closely related objection was already raised by Ben-Yami (1993), who argued that the Chinese Room cannot adequately simulate the ability of ordinary computational systems to determine and refer to the current time. This highlights a fundamental limitation: either the room must possess some form of internal temporal tracking (thereby going beyond pure syntactic symbol manipulation), or it will fail to handle ordinary temporal indexicals in a way that would be indistinguishable from a competent speaker.

At first glance, one might think that sufficiently sophisticated instructions could solve the problem. Perhaps the rulebook could contain different answers corresponding to different conversational contexts. In principle, one could even imagine an enormous branching library encoding possible dialogue trajectories. Yet this strategy quickly becomes implausible. The duration of the conversation may be arbitrary, the pauses between questions may vary unpredictably, and the possible combinations of exchanges may grow combinatorially without bound. More fundamentally, the system lacks any genuine temporal anchor. Searle has no clock, no sensory access to environmental change, and no independent mechanism for tracking lived duration. Once again, the system appears to require something close to an omniscient anticipation of all possible future conversational developments. Nevertheless, in order to appear rational in unrestricted conversation, he must be capable of distinguishing ten minutes from ten days, comparing temporal intervals, and responding coherently to questions concerning the temporal structure of the exchange itself.

The broader point is therefore analogous to the one raised by perceptual indexicals. Either the Chinese Room lacks the resources necessary to answer temporally indexed questions in a plausibly Turing-indistinguishable manner, or additional mechanisms for temporal tracking and contextual updating must be introduced into the system. But once such mechanisms are added, the room ceases to look like a purely syntactic device operating independently of pragmatic and world-related constraints. The original Chinese Room preserves the anti-functionalist intuition only insofar as it remains radically detached from perception, embodiment, and temporal self-location; yet precisely this detachment makes it doubtful that it could sustain unrestricted Turing-style interaction in the first place.

Searle directly addressed an objection of this kind in the 2015 interview (Searle, 2015, p. 53–54). We suggested that the Chinese Robot is not equivalent to the original Chinese Room precisely because the robot possesses sensors and systematic correlations between perceptual inputs and Chinese symbols. Searle rejected this interpretation. According to his reply, the person inside the robot receives only symbols, exactly as an ordinary processor does. External objects may causally produce those symbols, but the central processor itself learns nothing about those causal relations. In his view, the addition of sensors changes nothing philosophically important: the robot merely extends the chain of syntactic symbol manipulation, while genuine semantic understanding still never appears.

However, this response arguably underestimates the epistemic consequences of systematic sensorimotor correlation. Suppose Searle repeatedly receives one class of symbols whenever a red Coca-Cola can is placed before the camera, another when the object is removed, and yet another when the can is rotated or moved closer. Even if he initially manipulates these symbols blindly, prolonged participation in such regularities makes at least partial interpretive learning highly plausible. The issue is not that Searle suddenly becomes an ordinary Chinese speaker, but that the original intuition of total semantic opacity begins to erode. Once symbols are reliably connected to stable perceptual patterns, the system no longer resembles a purely formal calculus detached from the world.

More significantly, Searle's reply appears to shift the burden of the argument. The original Chinese Room derives much of its intuitive force from the claim that a human agent can execute the entire program and operate with all input information while remaining completely ignorant of meaning. But the Chinese Robot introduces an architecture in which successful performance depends precisely on dynamic relations between symbols and environmental states. If Searle is denied access to those relations, then the robot is no longer functionally equivalent to the original room, since crucial parts of the system become opaque even to the agent implementing it. But if he is granted such access, then at least partial semantic understanding becomes increasingly difficult to rule out a priori. The Chinese Robot, therefore, places Searle in a dilemma: either preserve the original intuition of semantic blindness at the cost of weakening the robot's Turing-level capacities, or preserve those capacities by introducing perceptual grounding that undermines the sharp separation between syntax and semantics on which the original argument depends. A defender of the Chinese Room may again respond, as in the case of iconic and quasi-iconic elements, by simply prohibiting the use of such indexicals in conversations with the room. However, as in the previous cases, such a restriction appears arbitrary and would substantially diminish both the linguistic competence of the room and its ability to plausibly pass an unrestricted Turing Test.

### 3.3 Symbols

We now turn to symbolic signs. It is important to note that the word “symbol” is being used here in a different sense from the one usually employed in discussions of the Chinese Room. For Searle, “symbols” are simply formal signs manipulated according to syntactic rules. In the Peircean framework, by contrast, a symbol is a specific kind of sign whose connection to its object and to other signs is mediated through convention, habit, and what Peirce calls Thirdness. We do not claim that Peirce's classification maps perfectly onto all the cases discussed below. For our purposes, it functions only as a convenient organizing framework, and the notion of “symbol” employed in this section should therefore be understood in this quasi-Peircean sense. What matters is that the following cases involve rule-governed linguistic practices that an ordinary machine can handle, but that Searle cannot easily allow himself to handle without either acquiring some understanding or restricting the conditions of the test.

The Chinese Room argument presupposes a deep separation between syntax and semantics, and this assumption is itself problematic. Both natural and formal languages possess a kind of intrinsic syntactic semantics: structural meanings generated by word order, grammatical constructions, and formal markers independently of lexical content. For example, the difference between ‘the mother loves the daughter' and ‘the daughter loves the mother' is produced entirely syntactically. In Chinese, a highly analytic language, this phenomenon is even more pronounced. Of course, the person inside the Chinese Room is supposed merely to manipulate symbols according to formal rules, and syntactic semantics alone would not immediately grant genuine understanding. Nevertheless, it is quite plausible that prolonged engagement with the system would allow the person to notice certain regularities — such as sentence-final particles and recurrent grammatical patterns — and thereby begin to develop an intuitive sense of construction types and syntactic boundaries. Such sensitivity would still fall far short of full semantic understanding and might well be too weak for Searle to find it problematic. However, this line of criticism gains considerable force when combined with the pragmatic dimensions of language use discussed below.

The first case concerns questions about the dialogue itself. The examiner may ask: “How many times did I mention the boy's name?”, “How many sentences were there in my previous reply?”, or “What did I refer to when I used the word ‘that'?” Such questions require the system not merely to produce isolated answers, but to track the structure of the ongoing conversation, identify repetitions, resolve anaphoric references, and perform metalinguistic operations on the dialogue. A machine can do this without difficulty: even simple text-processing systems can count words, locate repeated expressions, and search previous context. But for Searle, the situation is different. If he is instructed to scan previous Chinese strings and count occurrences of a given symbol, then he may well begin to grasp at least some elementary features of the dialogue. If, on the other hand, such questions are prohibited, then the Chinese Room becomes less conversationally competent than an ordinary machine.

The second case concerns language games. The examiner may introduce special rules for the exchange: for example, ‘answer only yes or no', ‘repeat the verb if the answer is affirmative', or ‘use only one-symbol replies'. In such cases, the meaning of the exchange is partly constituted by the explicit rule of the game. Machines can easily participate in such constrained linguistic practices. Searle, however, cannot safely do so. If he repeatedly produces one type of symbol in affirmative contexts and another in negative contexts, he may begin to infer that the contrast corresponds to affirmation and denial. If he is allowed to play enough such games, he may acquire fragments of semantic understanding precisely through the rule-governed use of symbols. But if he must refuse or block these games, then again the room no longer plausibly models unrestricted Turing-level linguistic interaction.

Thus, the symbolic cases reproduce the same dilemma encountered in the iconic and indexical cases. Either Searle permits ordinary rule-governed communicative practices, in which case the opacity of Chinese symbols becomes unstable; or he imposes artificial restrictions on the exchange, in which case the Chinese Room's ability to pass the Turing Test is correspondingly diminished.

### 3.4 Revisiting the Lazy Hume Planet and the Searle demon

The preceding discussion also allows us to return briefly to two scenarios introduced in the historical section: the Lazy Hume Planet and the Searle demon.

At first sight, the Lazy Hume Planet seems to strengthen the anti-functionalist intuition: it is hard to believe that a slow, spatially dispersed population could instantiate Hume's conscious reflection on induction merely by reproducing the relevant functional organization. But part of the force of this intuition may derive from the way the planet is imagined: as practically inert, communicatively isolated, and detached from ordinary engagement with the world. Suppose, however, that the planet does not merely simulate Hume's brain in silence, but very slowly communicates with us, explains Hume's theory of induction, answers objections, corrects misunderstandings, and situates its claims within an extended exchange. The case would then look different. It would no longer be merely an opaque functional duplicate considered from the outside, but a system participating, however slowly, in a temporally extended and pragmatically structured communicative practice. This would not prove that the planet is conscious. Yet it helps explain why a functionalist might follow Chalmers in biting the bullet: once the relevant functional organization is embedded in sufficiently rich communicative and world-involving relations, the original anti-functionalist intuition becomes less secure.

The Searle demon case can be read in the same light, but from the opposite direction. A demon that merely transmits signals between neurons is not itself a subject of understanding; it is only a component within a larger world-involving cognitive system. The reason the Chinese speaker in that scenario still appears to understand Chinese is not that each internal causal intermediary understands anything, but that the organized person as a whole remains embedded in perception, memory, action, and ordinary linguistic practice. From the Peircean perspective, the relevant system is not a bare chain of formal transitions but a sign-using organism whose symbols are connected with iconic, indexical, conventional, and pragmatic relations. This is precisely what the original Chinese Room removes. The more radically a functional system is isolated from ordinary communicative embedding, the stronger the anti-functionalist intuition becomes; the more we restore the pragmatic conditions of language use, the less stable that intuition is.

## 4 The Chinese token-training room

It is important to note that the Chinese Room argument does not straightforwardly apply to contemporary artificial intelligence systems. Searle's original argument was developed primarily against the background of symbolic AI — that is, approaches according to which intelligent cognition consists in the rule-governed manipulation of explicitly represented symbols according to syntactically specifiable programs. The very structure of the Chinese Room presupposes such a conception: there exists a determinate “program for speaking Chinese,” and the person inside the room executes this program by following formally describable instructions. However, many contemporary AI systems no longer operate in this manner. Neural-network-based architectures, including large language models, do not typically contain anything resembling a transparent rulebook or a syntactically interpretable program for conducting conversation. Their behavior emerges from distributed statistical structures acquired through training on extremely large datasets rather than from the explicit execution of hand-coded symbolic rules.

The question, then, is whether the dilemma developed in the previous sections reappears in the case of contemporary LLMs. If linguistic competence requires iconic, indexical, temporal, pragmatic, and world-involving dimensions of use, then a purely token-distributional analog of the Chinese Room will face the same problem as Searle's original room: either it remains isolated from these dimensions and fails to model unrestricted linguistic interaction, or it is enriched with them and thereby loses the intuition of total semantic blindness.

Nevertheless, contemporary AI systems are frequently subjected to criticisms structurally similar to Searle's. One of the best-known examples is the “stochastic parrot” critique (Bender et al., 2021). This critique should not be understood merely as the claim that large language models are statistical systems. The more important claim concerns the relation between linguistic form and meaning. Bender and Koller argue that systems trained only on linguistic form — that is, on observable strings and their distributional relations — do not thereby acquire access to meaning, since meaning involves more than relations among forms. On their view, meaning involves relations between linguistic forms, conventional meanings, communicative intents, and extra-linguistic situations (Bender and Koller, 2020). Bender et al. develop a related worry by arguing that language-model output may be only seemingly coherent: LMs produce sequences of linguistic forms on the basis of probabilistic information about how such forms combine, while human readers tend to interpret those outputs as meaningful by attributing communicative intent and coherence to them. On this view, the problem is not simply that LLMs predict tokens, but that their apparent linguistic competence is acquired from linguistic form detached from the communicative and world-involving conditions under which meaning is normally produced and interpreted.

It is possible to construct a Chinese Room-style argument against the claim that LLMs understand language, but the analogy must be formulated carefully. Modern LLMs are not trained primarily through explicit correction, approval, or disapproval in the manner of a human pupil being guided by a teacher. In the central pretraining phase, GPT-style language models are trained on a self-supervised next-token prediction objective: given a preceding sequence of tokens, the model is optimized to assign high probability to the next token in the training corpus. Later post-training procedures, including supervised fine-tuning and reinforcement learning from human feedback, may shape instruction-following, helpfulness, harmlessness, and conversational style, but these procedures do not constitute the whole or even the primary source of the model's linguistic competence.

With this qualification in place, we may formulate a Chinese Token-Training Room. Imagine a person locked in a room. He does not understand Chinese. He cannot speak Chinese, read Chinese, or translate Chinese into any language he knows. Inside the room, there is a terminal. At each stage, he is shown a sequence of Chinese tokens with the final token removed, and he must predict the missing continuation. He is not told what the sentence means. He is not shown objects, pictures, speakers, gestures, or situations. No teacher explains the meaning of the signs in a language he understands. He receives only feedback concerning predictive success: which Chinese token actually occurred next, and perhaps how far his prediction was from the expected continuation in the corpus.

Over an enormous number of such episodes, the person becomes extremely good at prediction. He learns that some tokens tend to follow others, that certain long-range dependencies matter, that different genres and registers license different continuations, and that question-like sequences are often followed by answer-like sequences. He becomes sensitive to recurring grammatical patterns, stylistic features, discourse roles, and forms of textual coherence. If a later alignment stage is added, he may also learn that some continuations are preferred by human raters and others are dispreferred. But the primary mechanism remains predictive adaptation to patterns in Chinese token sequences.

Eventually, he develops an extraordinarily complex set of dispositions. He does not consult a book of rules. In a sense, the “rules” are now embodied in him: his habits, expectations, and internal sensitivities have been shaped by a massive history of token-prediction training. Suppose now that, after this training, he is asked to produce longer Chinese continuations in response to prompts. From the outside, his answers are impressive. They are coherent, responsive to context, and often factually appropriate. He can explain poems, solve problems, summarize stories, imitate styles, answer objections, and even describe what it is like to understand Chinese.

But the proponent of a Chinese Room-style stochastic-parrot argument would insist that, from the inside, nothing has changed in the crucial respect. The man still does not understand Chinese. He has not moved from meaninglessness to meaning. He has only become extremely skilled at predicting and producing Chinese token continuations. The training process replaces the rulebook: instead of consulting explicit syntactic instructions, the subject has internalized an immense pattern of probabilistic expectations. The resulting behavior may be fluent and appropriate, but, according to the objection, appropriateness of token production is not the same as understanding.

The point of this thought experiment is not that a single human being could literally instantiate a transformer architecture, gradient descent, high-dimensional embeddings, attention heads, or distributed parameter updates. The analogy is deliberately idealized. It is intended to isolate the epistemic structure emphasized by stochastic-parrot-style objections: a system may become extremely good at producing contextually appropriate continuations by being trained on linguistic form, while never being given independent access to non-linguistic grounding or communicative situations. The Chinese Token-Training Room should therefore not be understood as a technical simulation of LLM training, but as a philosophical reconstruction of the claim that token-predictive competence, even when enormously sophisticated, may fall short of understanding.

This argument is deliberately more modest than the original Chinese Room argument. It does not assume that LLMs contain no internal structure. Indeed, recent work on probing, linear representations, and mechanistic interpretability suggests that models trained on prediction objectives can acquire highly structured internal representations. Probing work has provided evidence that syntactic structure can be recovered from neural representations, for example through structural probes of contextual embeddings (Hewitt and Manning, 2019). Other studies suggest that models can encode semantic or evaluative features in relatively low-dimensional or linear directions, as in work on sentiment and truth-related representations (Tigges et al., 2023; Burns et al., 2022; Marks and Tegmark, 2023). More directly relevant to the question of world-like internal structure, work on sequence models trained on games has found evidence of internal representations of board states, while recent work on LLMs suggests that spatial and temporal information may also be represented in activation space (Li et al., 2022; Nanda et al., 2023; Gurnee and Tegmark, 2024). Mechanistic interpretability work on factual associations likewise suggests that at least some model behaviors depend on structured and partially localizable internal computations rather than on mere surface association (Meng et al., 2022). Such findings do not by themselves establish genuine understanding. But they do show that the inference from “trained by next-token prediction” to “contains no meaningful internal representations” is too quick. A serious Chinese Room-style argument against LLM understanding must therefore be directed not against the existence of internal representations as such, but against the claim that the kinds of representations produced by token-predictive training are sufficient for semantic understanding.

The idea of extending the Chinese Room to large language models is not entirely new. Khamassi, Nahon, and Chatila have proposed a related thought experiment, the Chinese Room with a Word Transition Dictionary. Their proposal, however, differs from the Chinese Token-Training Room both in its internal organization and in the question it is meant to address. Whereas their discussion concerns the limitations of purely statistical systems with respect to strong alignment, our concern is understanding. Furthermore, their subject relies on an external transition dictionary, whereas ours acquires its dispositions through token-prediction training (Khamassi et al., 2024). Our thought experiment is structurally closer to Harnad's Chinese/Chinese dictionary-go-round: a subject who has access only to Chinese symbols can move indefinitely from one symbol string to another without ever reaching grounded meaning (Harnad, 1990). A literary analog of this structure can be found in Stanisław Lem's *The Star Diaries*, where Ijon Tichy searches an encyclopedia for the meaning of “sepulkas” and is sent from “sepulkas” to “sepulcaries,” from “sepulcaries” to “sepulation,” and finally back to “sepulkas.”

However, the stochastic-parrot critique has itself been refined and challenged in recent literature. Some authors argue that LLMs may acquire genuine symbols, structured internal representations, or nontrivial forms of grounding through training on language alone or through interaction with users and environments (Pavlick, 2023; Mandelkern and Linzen, 2024). Others propose more nuanced intermediate positions between the claim that LLMs understand nothing and the claim that they possess full human-like comprehension (Mitchell and Krakauer, 2023; Borg, 2025). Particularly useful here is the distinction between formal linguistic competence and functional linguistic competence: LLMs may be highly proficient at the former, while still lacking or only partially possessing the latter. Formal linguistic competence concerns the ability to model grammatical, lexical, and textual patterns. Functional linguistic competence concerns the use of language in situated, goal-directed, socially accountable, and world-involving activity. This distinction allows us to avoid a false dilemma. The issue is not whether LLMs are either empty parrots or fully understanding subjects. The more plausible question is which kinds of linguistic and cognitive competence they possess, and whether those competences are sufficient for semantic understanding.

The dialectical upshot is not that LLMs possess semantic understanding. It is that once a system is credited with capacities that go beyond mere distributional sensitivity — capacities involving context, pragmatic use, temporal tracking, or world-involving interaction — the simple stochastic-parrot dismissal requires further argument. The critic must then explain why these further capacities are still insufficient for understanding.

This point also connects with recent methodological critiques of LLM evaluation. Li et al. (2026) argue that much work on LLM psychology risks reproducing a form of behaviorism: it infers psychological or cognitive capacities from observable input-output regularities while leaving internal mechanisms theoretically underdescribed. This concern is compatible with our argument. We do not claim that unrestricted linguistic behavior is by itself sufficient to establish understanding or consciousness. Rather, our claim is negative and dialectical: if a system's linguistic performance genuinely involves capacities beyond mere distributional sensitivity, then the simple stochastic-parrot dismissal is no longer adequate. The critic must then either show that these further capacities are only behavioral artifacts, or provide an account of why they remain insufficient for understanding.

A related methodological issue arises in prompt-driven agent systems. Li and Wu (2025) argue that generative agent-based models face a dilemma between control and behavioral authenticity: stronger prompt constraints may improve reliability and internal validity, but they can also predetermine the very behaviors that are later interpreted as emergent. This dilemma parallels the problem faced by the Chinese Token-Training Room. If the interaction is heavily constrained, the system may preserve the appearance of mere token manipulation, but only by failing to model unrestricted linguistic practice. If, by contrast, the system is allowed to participate in richer, less artificially constrained forms of interaction, then its behavior can no longer be dismissed simply as the output of a narrow token-distributional mechanism. The methodological challenge is then to distinguish genuine pragmatic competence from artifacts introduced by the experimental or prompt structure.

The Chinese Token-Training Room therefore faces the same dilemma as the original Chinese Room. If the room is restricted to bare token-distributional training, it preserves the intuition that the trained subject lacks understanding: the subject receives only Chinese tokens and predictive feedback, and gradually learns which continuations are more or less expected. But under these restricted conditions, it is doubtful that the system could sustain genuinely unrestricted linguistic interaction, because full-fledged linguistic exchange requires more than statistically appropriate continuation. It involves sensitivity to context, indexical and temporal features of interaction, pragmatic norms, communicative roles, and often the surrounding world.

If, however, the room is enriched with the resources required for unrestricted interaction, the intuition of total semantic blindness is weakened. If certain symbols are reliably connected with recurring perceptual patterns, temporal changes, conversational roles, practical consequences, or socially structured feedback, the subject's activity no longer looks like mere manipulation of uninterpreted tokens. The system begins to approximate the conditions under which meaningful linguistic competence is normally acquired and exercised. Our claim is not that statistical mechanisms automatically yield understanding, nor that passing an unrestricted Turing Test would be sufficient for phenomenal consciousness. The point is more limited: sufficiently rich pragmatic capacities would weaken the simple inference from token-predictive training to absence of understanding.

There is, however, a further objection to this line of thought. One might argue that behavioral evidence, even when rich and flexible, should carry less weight in the case of AI systems than in the case of nonhuman animals. AI systems are extremely good at behavioral tests; in some domains, they already outperform nonhuman animals and even humans. Yet, unlike nonhuman animals, they do not share our biological nature. In the animal case, behavioral evidence is significant partly because we assume a relevant continuity between human and nonhuman biological systems (Birch, 2022; Birch et al., 2020). In the AI case, this common biological background is absent. Moreover, AI systems may be especially good at gaming test parameters: producing the right outputs without possessing the underlying capacities we are trying to detect.

This objection is important, but it should be carefully limited. It is strongest when directed against the claim that behavioral evidence alone could establish consciousness. In the case of AI systems, we probably need a more theory-heavy approach: we should examine not only behavior, but also the computational architectures proposed by theories of consciousness and ask whether those architectures, or their essential features, are realized in AI systems or could be realized in them (Butlin et al., 2025). In this respect, AI consciousness may require a different evidential strategy from animal consciousness. This is why our appeal to unrestricted linguistic interaction should not be read as a return to a purely behaviorist criterion. Behavioral evidence may be important, but in the case of AI it must be combined with theory-driven analysis of architecture, internal mechanisms, grounding, and the role of experimental or prompt-induced constraints.

However, this does not show that linguistic behavior is irrelevant to the assessment of AI understanding. Full-fledged linguistic communication is not a shallow behavioral capacity. It plausibly requires contextual sensitivity, temporal tracking, pragmatic responsiveness, stable conversational roles, and some form of world-directed or action-guiding competence. These are precisely among the central challenges faced by current LLMs and LLM-based agents (Chalmers, 2023; Bennett, 2023). Thus, if an LLM-based system were able to sustain unrestricted, temporally extended, context-sensitive, and pragmatically embedded linguistic interaction, this would not settle the question of consciousness, nor would it show that the system understands language in exactly the way humans do. But it would substantially weaken the simple stochastic-parrot dismissal. At minimum, it would show that the system is not merely producing locally plausible token continuations, but is capable of participating in a broader pragmatic, temporal, and world-involving practice of language.

The resulting position is therefore intermediate. Behavioral evidence should not be treated as sufficient on its own to establish either understanding or consciousness in AI systems. But neither should it be dismissed as irrelevant. Unrestricted linguistic competence is not just one behavioral capacity among others. It involves the integration of context-sensitive, temporally extended, pragmatically structured, and socially embedded forms of interaction that are directly relevant to the assessment of understanding. Its bearing on consciousness remains more indirect and theory-dependent.

The moral of both the original Chinese Room and the Chinese Token-Training Room is therefore methodological. A radically isolated symbol-manipulating system may fail to understand, but it may also fail to model unrestricted linguistic competence. Conversely, a system capable of genuinely unrestricted linguistic interaction cannot be characterized merely as a device for producing locally plausible strings, unless the critic gives an additional argument explaining why its integrated pragmatic, temporal, contextual, and world-involving capacities still fall short of understanding. Thus, the Chinese Token-Training Room does not show that LLMs understand language. Rather, it shows that the stochastic-parrot objection, like Searle's original Chinese Room, must either restrict the system in ways that make unrestricted linguistic competence doubtful or allow forms of pragmatic embedding that weaken the intuition of total semantic opacity.

## 5 Conclusion

In this paper, we argued that the force of Searle's Chinese Room depends on an artificially impoverished conception of linguistic interaction. Ordinary language use is permeated by iconic, indexical, symbolic, and pragmatic structures that cannot easily be excluded without substantially weakening the room's ability to sustain unrestricted Turing-style communication. Once such structures are introduced, the intuition of total semantic opacity becomes increasingly unstable. We further argued that similar considerations apply not only to classical symbolic AI, but also to contemporary large language models and “stochastic parrot” objections. If a system were genuinely capable of sustaining unrestricted, context-sensitive, temporally extended, and pragmatically embedded linguistic interaction, this would not by itself establish genuine understanding, still less phenomenal consciousness. It would, however, make it harder to dismiss the system a priori as a case of mere syntactic manipulation or local token prediction, since unrestricted linguistic interaction is an integrative capacity involving forms of contextual sensitivity, temporal tracking, pragmatic responsiveness, and social embedding that are directly relevant to the assessment of understanding. Its bearing on consciousness would remain more indirect and theory-dependent. None of this establishes the truth of functionalism or shows that successful linguistic performance is sufficient for consciousness. However, it does suggest that neither the original Chinese Room nor its token-training analog succeeds in demonstrating that functional or computational organization is in principle insufficient for genuine understanding.

## Data Availability

No original data were generated or analyzed in this study.
